# Rapidly progressive Bronchiolitis Obliterans Organising Pneumonia presenting with pneumothorax, persistent air leak, acute respiratory distress syndrome and multi-organ dysfunction: a case report

**DOI:** 10.1186/1752-1947-2-145

**Published:** 2008-05-06

**Authors:** Indranil Chakravorty, William LG Oldfield, Carlos MH Gómez

**Affiliations:** 1 Department of Respiratory Medicine, Lister Hospital, Beds & Herts Postgraduate Medical School, Stevenage, UK; 2 Adult Intensive Care Unit, St Mary's Hospital, London, UK

## Abstract

**Introduction:**

Bronchiolitis Obliterans Organising Pneumonia (BOOP) may often present initially as a recurrent spontaneous pneumothorax and then develop multi-system complications.

**Case presentation:**

A 17-year-old boy presented with a pneumothorax, which developed into rapidly progressive Bronchiolitis Obliterans Organising Pneumonia (BOOP). He developed multi-organ dysfunction (including adult respiratory distress syndrome, oliguric renal failure, acute coronary syndrome, cardiac failure and a right atrial thrombus) which necessitated prolonged intensive care. Diagnosis was confirmed on open lung biopsy and he responded well to treatment with corticosteroids.

**Conclusion:**

BOOP is exquisitely sensitive to oral corticosteroids but if the diagnosis is not considered in such patients and appropriate treatment instituted early, BOOP may often lead to prolonged hospital admission with considerable morbidity.

## Introduction

**Bronchiolitis Obliterans Organising Pneumonia (BOOP) **is a clinico-pathological entity [1] characterised histologically by polypoid masses of granulation tissue in the lumen of small airways, alveolar ducts and alveoli co-existing with fibrosis [2]. Its distribution is patchy, with preservation of background architecture [1]. Lung function tests demonstrate impaired diffusion capacity with a restrictive picture [3] while the radiological appearance is of patchy consolidation and alveolar infiltrates in a peri-bronchiolar or pleural distribution [4]. We report on what we believe to be the first case of BOOP associated with persistent air leak, multiple organ failure and acute coronary syndrome.

## Case presentation

A 17-year-old Asian man presented with a spontaneous pneumothorax, which was treated initially by needle aspiration. It recurred within a week with complete right-sided pneumothorax requiring water-sealed intercostal drainage. The air leak persisted after one week and the patient developed pyrexia with associated neutrophilia and raised serum inflammatory markers. A chest radiograph showed a hydro-pneumothorax and culture of the pleural fluid grew *Pseudomonas aeruginosa *and *Methicillin-resistant Staphylococcus aureus (MRSA)*. He was treated with intravenous Piperacillin-Tazobactam resulting in a full clinic-radiological recovery.

A week later his fever returned and repeat chest radiograph showed right middle and lower lobe consolidation associated with recurrent hydro-pneumothorax. A new intercostal drain was inserted and he was intubated and ventilated due to the rapid onset of severe respiratory distress. Computed tomogram (CT scan) of the chest confirmed bilateral patchy consolidation in association with widespread ground-glass opacities, sub-pleural cavitation and bilateral pleural effusions. He underwent an open thoracotomy, which did not reveal any macroscopic evidence of an organised or loculated empyema.

Postoperatively he developed multi-organ dysfunction with oliguric renal failure, prolonged mechanical ventilation, consumption coagulopathy, elevated amino-transaminases and an anteroseptal myocardial infarction with moderate left ventricular systolic dysfunction as evidenced by transoesophageal echocardiography (TOE) and raised troponin I.

Multiple sampling of blood, urine and bronchoscopic lavage fluid for culture did not show any new bacterial, fungal or viral infection and the leukocyte count remained normal. Serological testing for atypical pneumonia (*Legionella, Mycoplasma, Chlamydia and Coxiella*), viral infection (*Hepatitis virus A, B & C, Cytomegalovirus, Herpes viruses*), and human immunodeficiency virus was negative. His autoimmune screen was negative (Anti-nuclear antibody, Rheumatoid factor and Anti-cytoplasmic antibodies).

He was treated with broad-spectrum antibiotics (*Amoxycillin with Clavulinic acid, Clarithromycin, Piperacillin-Tazobactam, Gentamicin and Vancomycin*) and an anti-fungal (*Voriconazole*) for four weeks without any clinical or radiological improvement.

He developed a right-sided broncho-pleural fistula complicated by pneumo-mediastinum and pneumo-pericardium, widespread consolidation, alveolar infiltrates, pulmonary haemorrhage and bilateral pleural effusions. He deteriorated again in his 4^th ^week with pyrexia and increasing inotrope dependence. A TOE confirmed persistent poor left ventricular function and a right atrial thrombus. He underwent median sternotomy and cardiopulmonary bypass to enable removal of the thrombus, at which time the opportunity was taken to perform an open lung biopsy from the right middle lobe. This showed changes showing alveolar exudates consistent with BOOP in association with patchy pulmonary haemorrhage and alveolar exudate.

He was commenced on corticosteroids (*Prednisolone 1.5 mg/kg*) with improvement clinically and radiologically within 72 hours. This was manifested by a reduction in oxygen requirement, reduced inflammatory markers, resolution of fever and disappearance of radiographic infiltrates.

Two weeks later, he was transferred to level II care for further weaning and rehabilitation. He represented three months later with a recurrent right-sided pneumothorax and underwent a pleurectomy. Subsequently, there have been no recurrences and the corticosteroid therapy has been rapidly weaned.

## Discussion

BOOP may be idiopathic (which is associated with a better prognosis) [5] or secondary to bacterial (*Mycoplasma*) or viral infections (*Human immunodeficiency virus, Herpes simplex virus*), pharmacological agents (*Nitrofurantoin*, *Sulfasalazine*) [6], chemotherapy, radiotherapy [7] and connective tissue disorders.

Characteristic CT appearances in conjunction with broncho-alveolar lavage [8] finding of relative neutrophilia and reduction in the CD4/CD8 ratio, is suggestive of BOOP. Transbronchial [8] and CT guided percutaneous approaches have a poor yield due to the patchy distribution of BOOP and small sample size. The differential diagnosis includes Acute Interstitial Pneumonitis and respiratory distress syndrome both of which may be associated with pneumonia or BOOP [9], as well as all the causes of cardiogenic pulmonary oedema. In patients who present with fulminant widespread consolidation with alveolar infiltrates, treatment is usually commenced empirically after collection of microbiological specimens.

However the potential benefit of timely corticosteroid therapy in patients with BOOP may justify the added risk of an open lung biopsy [10,11] especially when obvious infective or cardiogenic causes cannot be identified. Patients with BOOP have a > 65% cure rate on corticosteroid therapy in most case series [1].

The initial sequence of events described in this case with a primary pneumothorax followed by a persisting air leak and signs of infection are not unknown. In our patient however, after an initial full clinico-radiological recovery, there was recurrence of the leak and development of a rapidly progressive 'acute inflammatory state' associated with multi-organ dysfunction, absence of infection and failure of broad-spectrum anti-microbials and an anti-fungal agent to improve clinical or radiological parameters. This was unusual and suggested an alternative pathology.

The second peculiarity of our case is the fulminant progression of BOOP into multi-organ failure after two weeks of apparent stability. A rapidly progressive type with multi-organ failure has indeed been described, albeit rarely [12]. Our patient, however, is to our knowledge the first reported case of cured or silent BOOP undergoing such conversion into a fulminant form.

This case highlights the importance of considering BOOP in the differential diagnosis of culture-negative respiratory failure in previously healthy patients. The *'Air Leak Syndrome' *type of BOOP is associated with persisting broncho-pleural fistula, pneumo-mediastinum and pneumo-pericardium and has been reported only rarely [13-15].

## Conclusion

To our knowledge the association in a previously healthy teenager of non-occlusive, Troponin I positive, acute coronary syndrome (with TOE confirmation of regional wall motion abnormalities but normal coronary arteries on CT angiogram as well as on direct surgical examination) with BOOP has not been hitherto described. In our patient appropriate treatment of the underlying left ventricular dysfunction did not alter the radiological appearances on serial high resolution CT scans. Although the likely explanation for the acute coronary syndrome in this patient may have been intravascular thrombosis there was no evidence of disseminated intravascular coagulation and, moreover, coagulation studies revealed raised prothrombin and thrombin times and thrombocytopenia with normal fibrinogen levels. The aetiology of acute coronary syndrome in association with BOOP remains unclear.

## Competing interests

The authors declare that they have no competing interests.

## Authors' contributions

All three authors were fully involved with the management of this case while in hospital and have equally contributed to the design, drafting and editing of the article submitted. The authors also acknowledge the contribution of other colleagues in the management of this challenging case.

## Consent

Written informed consent was obtained from the patient for publication of this case report and accompanying images.
